# Communications Between Pregnant Women and Maternity Care Clinicians

**DOI:** 10.1001/jamanetworkopen.2020.6636

**Published:** 2020-05-27

**Authors:** Erika R. Cheng, Aaron E. Carroll, Ronald E. Iverson, Eugene R. Declercq

**Affiliations:** 1 Indiana University School of Medicine, Division of Children's Health Services Research, Department of Pediatrics, Indianapolis; 2 Indiana University School of Medicine, Center for Pediatric and Adolescent Comparative Effectiveness Research, Indianapolis; 3 Boston University School of Medicine, Department of Obstetrics and Gynecology, Boston, Massachusetts; 4 Boston University School of Public Health, Department of Community Health Sciences, Boston, Massachusetts

## Abstract

This survey study assesses patients’ self-reported communication experiences with their maternity care clinicians and examines the association of these experiences with women’s reports of feeling pressure to have interventions during delivery.

## Introduction

Shared decision-making, when executed properly, is associated with increased patient satisfaction, improved health outcomes, and reduced health care costs.^1^ Health care lacking patient input may be associated with unwarranted practice variation and both overuse and underuse of health care. Effective clinician-patient communication may be associated with improved outcomes, but it requires patients to have an active and participatory role. One area that, to our knowledge, has not been explored is maternity care. We assessed patients’ self-reported communication experiences with their maternity care clinicians and examined associations of these experiences with women’s reports of feeling pressure to have interventions during delivery.

## Methods

This survey study used nationally representative data from Listening to Mothers III (LTM III),^2^ an online survey of 2400 English-speaking women (aged 18-45 years) who delivered a single infant in a US hospital between July 1, 2011, and Jun 30, 2012. Data analyses were conducted in June 2019. The Indiana University School of Medicine deemed this study to be exempt from institutional review board review because the data are publicly available. This study followed the American Association for Public Opinion Research (AAPOR) reporting guideline.

We examined a series of questions, including, “During a prenatal visit in your most recent pregnancy, did you ever hold back from asking questions or discussing your concerns because…?” We compared women’s responses to with their report of feeling pressured from the clinician to accept an epidural, labor induction, or cesarean delivery.

We examined women’s report of holding back from asking questions by sociodemographic and prenatal characteristics using χ^2^ tests. Statistical significance was set at 2-sided *P* < .05. We evaluated associations of holding back with feeling pressure to have an intervention using logistic regression adjusting for clinician type and sex, maternal age, educational level, race/ethnicity, payer source, nativity, marital status, parity, and childbirth education class attendance. Analyses were weighted to be nationally representative and were conducted using survey procedures in SAS, version 9.4 (SAS Institute).^2^

## Results

Of the 2400 women in the sample, most were non-Hispanic white (1458 [55.0%]), US born (2233 [93.0%]), and married (1607 [60.4%]), and most received care from obstetrics and gynecology specialists (1734 [70.9%]) (Table). More than 41.0% of women (n = 984) reported that they held back from asking their clinician questions. Their perceived reasons included the clinician seemed rushed (29.6%), they wanted care that differed from the clinician’s preference (20.5%), and fear of being perceived as difficult (23.3%). Younger, nulliparous women receiving care from health care workers who were not obstetrics and gynecology specialists or from female clinicians were the most likely to hold back from asking question (Table). These women also frequently reported feeling discriminated against for various reasons. After adjustment, women who held back from asking questions were more than 5 times more likely to report feeling pressure to have an intervention (adjusted odds ratio, 5.3; 95% CI, 4.0-7.1).

**Table.** Characteristics of Women Who Reported Holding Back From Asking Questions

Characteristic	Women, No. (%)^a^		P value
	Total (N = 2400)	Held back from asking questions (n = 984)	
Type of maternity care clinician			
Obstetrics and gynecology specialist	1734 (70.9)	663 (37.1)	.001
Family medicine doctor	298 (13.4)	152 (50.8)	
Midwife	221 (9.9)	90 (46.0)	
Other	147 (5.7)	79 (55.2)	
Clinician sex			
Female	1490 (61.0)	650 (44.7)	.001
Male	910 (39.0)	334 (35.0)	
Age, y			
18-24	601 (31.8)	302 (50.1)	<.001
25-34	1336 (53.1)	553 (39.3)	
≥35	463 (15.1)	129 (27.1)	
Education al level			
High school or GED	466 (42.3)	180 (38.0)	.14
Some college or associate's degree	581 (18.4)	240 (41.2)	
College degree or greater	1353 (39.3)	564 (43.8)	
Race/ethnicity			
Non-Hispanic white	1458 (55.0)	557 (38.7)	.047
Non-Hispanic black	309 (15.3)	129 (39.1)	
Non-Hispanic other race	181 (6.5)	91 (54.4)	
Hispanic/Latina	452 (23.1)	207 (43.4)	
Payer source			
Private	1317 (46.9)	498 (39.4)	.52
Public or Medicaid	1018 (53.1)	455 (41.2)	
Nativity			
US born	2233 (93.0)	904 (40.4)	.29
Foreign born	167 (7.1)	80 (46.9)	
Married at the time of birth			
Yes	1607 (60.4)	613 (39.1)	.14
No	793 (39.6)	371 (43.5)	
Prior deliveries			
None	1144 (40.7)	469 (47.1)	<.001
≥1	1256 (59.3)	515 (36.3)	
Attended childbirth education classes			
Yes	919 (34.2)	451 (50.8)	<.001
No	1481 (65.8)	533 (35.7)	
Felt discrimination owing to race/ethnicity, culture, or language			
Never or sometimes	2270 (94.0)	879 (38.2)	<.001
Usually or always	130 (6.1)	105 (82.4)	
Felt discrimination owing to health insurance			
Never or sometimes	2252 (91.8)	868 (37.4)	<.001
Usually or always	148 (8.2)	116 (80.4)	
Felt discrimination owing to a difference of opinion			
Never or sometimes	2230 (90.8)	845 (36.5)	<.001
Usually or always	170 (9.3)	139 (83.7)	
Clinician used medical words you did not understand			
Never or sometimes	2098 (85.3)	802 (37.4)	<.001
Usually or always	302 (14.7)	182 (60.9)	
Clinician spent enough time with you			
Never or sometimes	446 (20.3)	340 (71.5)	<.001
Usually or always	1954 (79.7)	644 (33.1)	
Clinician answered questions			
Never or sometimes	323 (15.5)	250 (73.9)	<.001
Usually or always	2077 (84.5)	734 (34.8)	
Clinician encouraged discussion about health questions or concerns			
Never or sometimes	482 (23.3)	342 (67.4)	<.001
Usually or always	1918 (78.7)	642 (33.7)	

^a^ Sample sizes are unweighted. Percentages are weighted estimates.

## Discussion

Patients need authority to make choices about their care without feeling coerced.^3^ Similar to the situation in nonmaternity settings,^4^ we found that many pregnant women may be reluctant to engage in the decision-making process. A total of 41.0% of women reported holding back from asking their maternity care clinician questions, and that reluctance was associated with greater odds of reporting feeling pressured to have an intervention. In other work, women who perceived pressure from clinicians for labor induction or cesarean delivery were more likely to undergo these procedures regardless of medical indication.^5^

These cross-sectional data could not determine directionality of these associations and are subject to recall bias. Nevertheless, this was the first study, to our knowledge, to examine patient-held fears of voicing disagreement and being perceived as difficult as barriers affecting maternity care. Findings suggest a breakdown in communication that is associated with women not fully participating in their maternity care. This presents an opportunity for interventions focused on patient activation.^6^ Such efforts should encourage women to ask questions and also help clinicians create an environment for open communication. We believe that both are needed to make sure the preferences of women are considered.
